# Induction of phenotypic changes in HER2-postive breast cancer cells *in vivo* and *in vitro*

**DOI:** 10.18632/oncotarget.27679

**Published:** 2020-07-28

**Authors:** Anastasia Frank-Kamenetskii, Julia Mook, Meredith Reeves, Corinne A. Boulanger, Thomas J. Meyer, Lauren Ragle, H. Caroline Jordan, Gilbert H. Smith, Brian W. Booth

**Affiliations:** ^1^Department of Bioengineering, Clemson University, Clemson, SC, USA; ^2^Department of Biological Sciences, Clemson University, Clemson, SC, USA; ^3^Center for Cancer Research, National Cancer Institute, National Institutes of Health, Bethesda, MD, USA; ^4^CCR Collaborative Bioinformatics Resource, National Cancer Institute, National Institutes of Health, Bethesda, MD, USA; ^5^Advanced Biomedical Computational Science, Frederick National Laboratory for Cancer Research, Frederick, MD, USA; ^*^These authors contributed equally to this work

**Keywords:** breast cancer, cancer cell redirection, microenvironment, stem cells

## Abstract

The influence of breast cancer cells on normal cells of the microenvironment, such as fibroblasts and macrophages, has been heavily studied but the influence of normal epithelial cells on breast cancer cells has not. Here using *in vivo* and *in vitro* models we demonstrate the impact epithelial cells and the mammary microenvironment can exert on breast cancer cells. Under specific conditions, signals that originate in epithelial cells can induce phenotypic and genotypic changes in cancer cells. We have termed this phenomenon “cancer cell redirection.” Once breast cancer cells are redirected, either *in vivo* or *in vitro*, they lose their tumor forming capacity and undergo a genetic expression profile shift away from one that supports a cancer profile towards one that supports a non-tumorigenic epithelial profile. These findings indicate that epithelial cells and the normal microenvironment influence breast cancer cells and that under certain circumstances restrict proliferation of tumorigenic cells.

## INTRODUCTION

Tissue microenvironments are complex regions that consist of multiple cell types such as epithelial cells, adipocytes, fibroblasts, vascular endothelial cells, resident and transient immune cells, and somatic stem cells [1]. A range of intercellular signals is produced by each cell type and helps regulate cell growth, homeostasis, and supports normal development. These naturally occurring signals direct cell differentiation and potentially prevent tumor progression by producing anti-proliferative and apoptotic signals for abnormal cells. Irregularities in production of needed stimuli or pathways that control cell proliferation will lead to uncontrolled apoptosis, tumor formations, and cancer [2]. Several studies have demonstrated that abnormal cellular transformation could be repressed as long as cancer cells were restricted to a tumor-hostile location within the tissue, and that the normal microenvironment can suppress cancer formation by cellular signaling from the surrounding cells and matrix [3–5]. These results suggest that cancer cells are responsive to external stimuli and can even be reverted back to the wild-type tissue phenotype by the signaling from cells within a healthy environment. This phenomenon has been termed “cancer cell redirection” [6].

The mammary gland is an excellent system to study stem cells, microenvironments, and development as the vast majority of cellular growth and differentiation occurs during puberty [7]. The glandular epithelium undergoes vast proliferation and differentiation during puberty. Cyclic remodeling of the mammary epithelium occurs during every menstrual cycle. Additional glandular expansion occurs during pregnancy and lactation followed by massive cellular remodeling during involution, that returns the gland to near pre-pregnancy conditions. Using rodent models, it was discovered that when mammary epithelial cells were transplanted into a mammary fat pads of pre-pubescent female mice devoid of endogenous epithelium an entire functional mammary outgrowth could be recapitulated regardless of age or parity status of the transplanted cells [8–10]. When dispersed cell suspensions of mammary epithelial cells are used in these models the cells participate in the formation of new microenvironments allowing for the normal development of mammary outgrowths.

The deterministic capacity of normal mammary microenvironments has been demonstrated by the incorporation of non-mammary stem cells into the reforming microenvironments. Stem cells isolated from the central nervous system, bone marrow, testes, and embryonic stem cells (ESCs) have been introduced into reforming mammary microenvironments and adopted mammary epithelial phenotypes [11–14]. Lineage-traced daughter cells of the non-mammary stem cells participated in the normal development of mammary ductal trees and differentiated into luminal epithelial cells, myoepithelial cells, and milk protein-producing secretory epithelial cells during lactation [11–14].

## RESULTS

### Human HER2^+^ breast cancer cells are redirected *in vivo*

**Figure 1:**
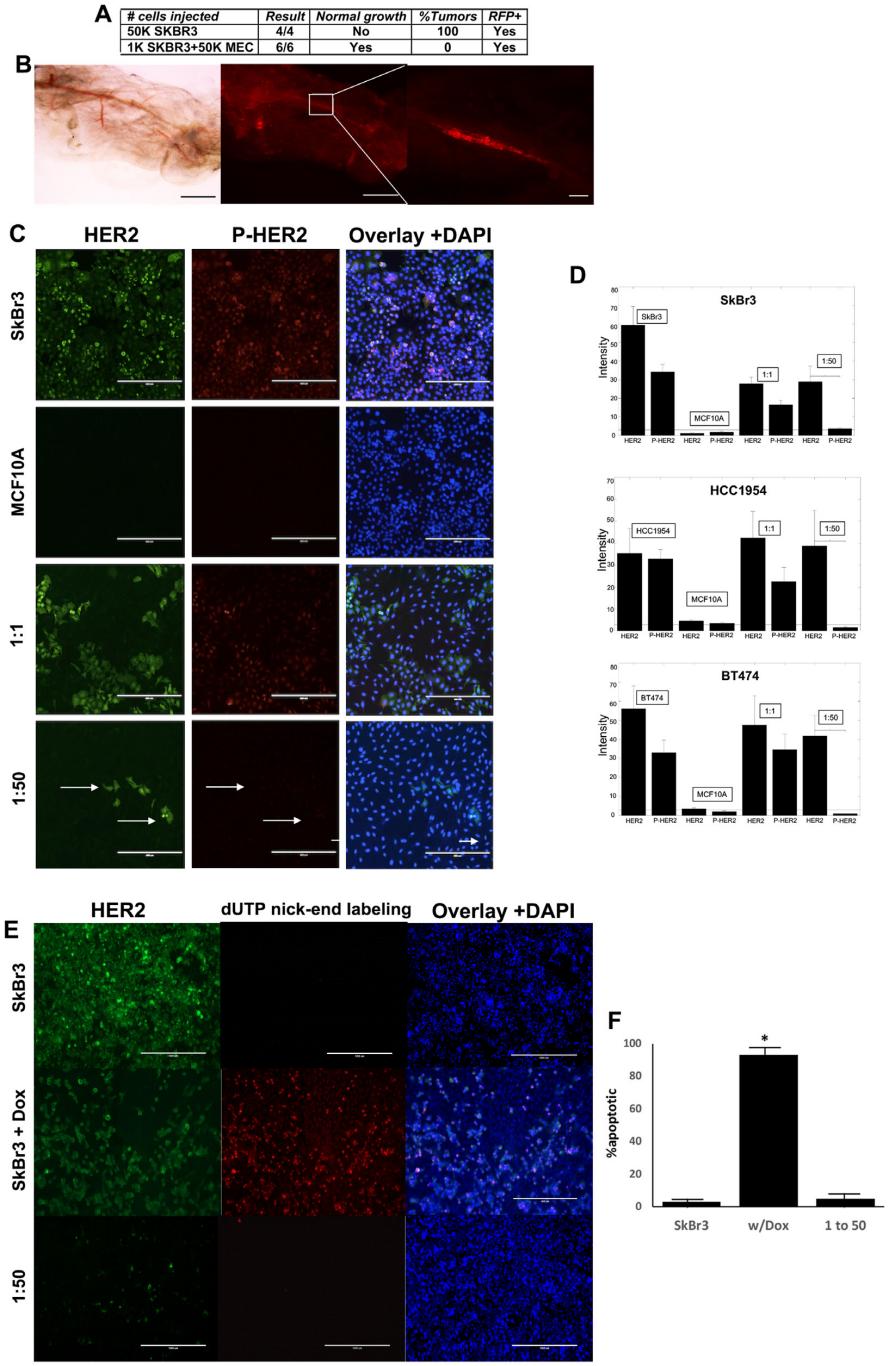
Human HER2^+^ breast cancer cells are redirected *in vivo* and *in vitro*. (**A**) Transplantation results of cancer cells alone or co-transplanted with MECs in a 1:50 ratio. (**B**) Mammary gland whole mount of mammary outgrowth comprised of RFP^+^ cancer cells and MECs after 9 weeks. Left-light image, middle-fluorescent image of left, Right-higher power image of box in middle. (**C**) IFC of SkBr3 and MCF10A cells grown alone or in co-cultures at the ratios indicated. Green-HER2, red-P-HER2, nuclei stained with DAPI. Scale bars = 100 μm. (**D**) Quantification of HER2 and P-HER2 expression. (**E**) Images of HER2 IFC (green) and TUNEL results (red), nuclei stained with DAPI. Scale bars = 100 μm. (**F**) Quantification of E.

It has been demonstrated previously that human triple negative breast cancer cells and human testicular carcinoma cells are redirected to adopt a mammary epithelial phenotype when co-transplanted with normal mammary epithelial cells (MECs) in a specific ratio of 1:50 (1 cancer cell for every 50 MECs) into cleared mammary fat pads of prepubescent female mice [4, 5]. We transplanted HER2^+^ breast cancer cells that constitutively express RFP (SkBr3-RFP) with and without MECs. When the HER2^+^ breast cancer cells were transplanted alone RFP^+^ mammary tumors formed in 100% of the recipient animals (4/4) (Figure 1A). However, when the HER2^+^ breast cancer cells were co-transplanted with MECs using the same 1:50 ratio no mammary tumors formed, and we found normal mammary ductal growth (Figure 1A and 1B). The resulting mammary ducts had RFP^+^ cells incorporated throughout the entire ductal trees (Figure 1B). Human HER2^+^ breast cancer cells are redirected to adopt a normal mammary epithelial phenotype in a similar fashion as erbB2^+^ mouse mammary cancer cells, human TNBC cells, and human testicular embryonal carcinoma cells *in vivo* [3–5].

### Human HER2^+^ breast cancer cells are redirected *in vitro*

We have developed and validated an *in vitro* model of cancer cell redirection using mouse mammary epithelial cells and mouse mammary tumor cells [6, 15, 16]. The mammary tumor cell lines redirected were derived from tumors that developed in MMTV-*neu* transgenic female mice. The MMTV promoter drives expression of the oncogene *neu* resulting in overexpression of erbB2 in the mammary glands of the mice. These tumors exhibit pathological similarities to human ER^-^/PR^-^/HER2^+^ breast tumors [3, 17]. Furthermore, following redirection, erbB2 remains overexpressed on the cell surfaces but signaling of erbB2 is attenuated [3, 6]. We use loss of receptor signaling as a biomarker of cancer cell redirection.

We introduced human breast epithelial cells (MCF10A cells) and human HER2^+^ breast cancer cells (SKBR3, BT474, HCC1954) into our *in vitro* model to assess the redirection capacity of human breast cancer cells. When HER2^+^ breast cancer cells were cultured alone they expressed both HER2 and phospho-HER2 indicating that the receptor was signaling (Figure 1C). Conversely, breast epithelial cells do not express detectable levels of HER2 or phospho-HER2 *in vitro* (Figure 1C). When the two cell types are co-cultured in equal numbers (1:1 ratio) the cancer cells continue to express both HER2 and phospho-HER2 (Figure 1C). However, when the two cell types are co-cultured using the redirection ratio of 1:50, the cancer cells continue to express HER2, but phosphorylation of the receptor is absent (Figure 1C, arrows). The reduction of HER2 phosphorylation was detected in all three HER2^+^ breast cancer cell lines used (SkBr3, BT474, HCC1954) (Figure 1D). This indicates that the HER2^+^ breast cancer cells have undergone phenotype redirection.

The question “Is apoptosis involved in cellular redirection” was addressed. HER2^+^ breast cancer cells were treated with doxorubicin and the results compared to untreated cancer cells and redirected cancer cells (Figure 1E and 1F). Doxorubicin induced apoptosis in the cancer cells, but very low levels of apoptosis were detected in untreated cancer cells and 1:50 co-cultures suggesting that apoptosis is not a major factor in cancer cell redirection *in vitro*.

### *In vitro* redirection induces phenotype changes

**Figure 2:**
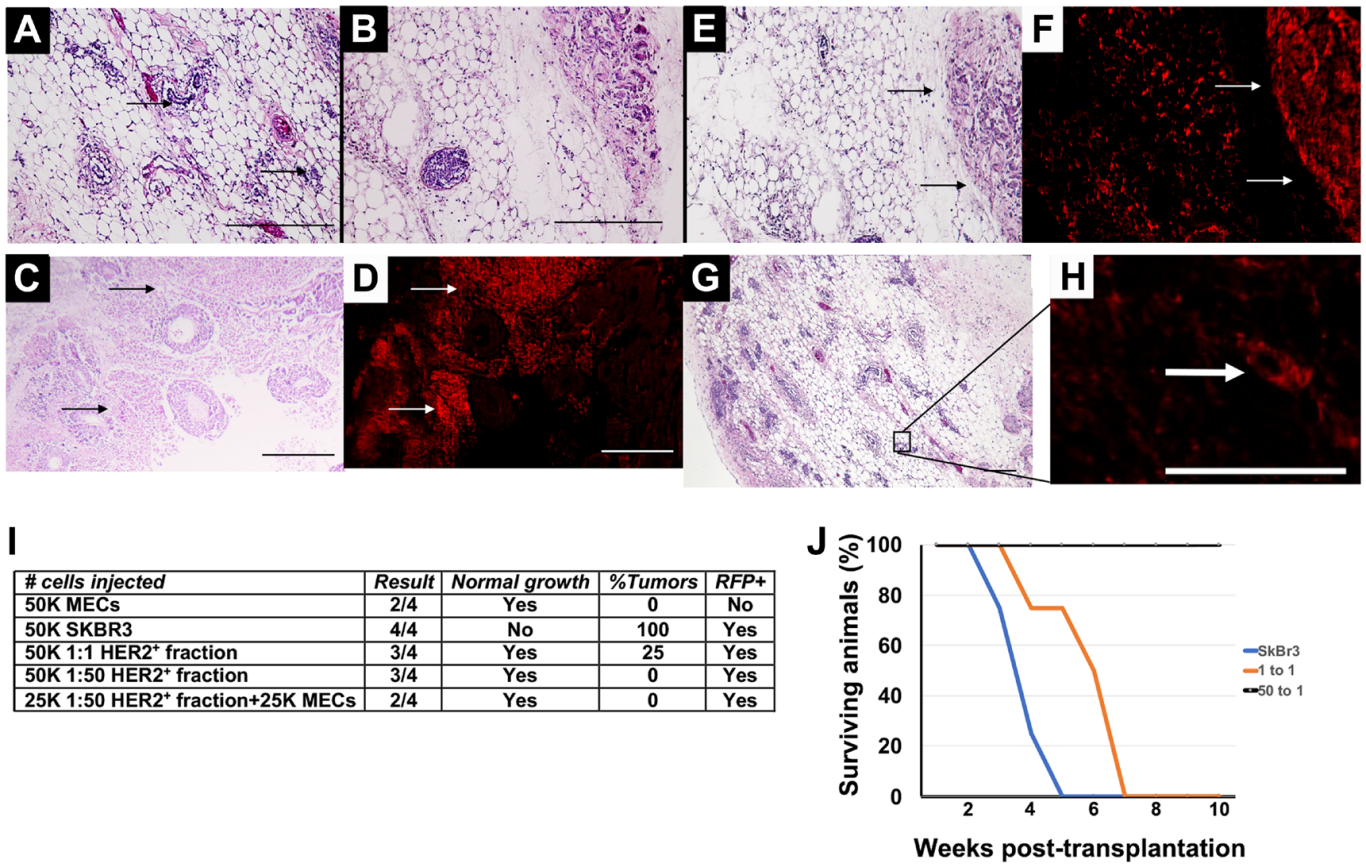
Transplantation results following *in vitro* redirection. (**A**, **B**) H&E staining of mammary outgrowth following MEC transplantation. (**C**) H&E staining of mammary tumor that formed following transplantation of SkBr3-RFP cells. (**D**) Fluorescent image of C. (**E**) H&E staining of mammary outgrowth and mammary tumor following transplantation of HER2^+^ 1:1 fraction. (**F**) Fluorescent image of E. (**G**) H&E staining of HER2^+^ 1:50 fraction. (**H**) Fluorescent image of outlined area in G. (**I**) Transplantation results. (**J**) Survival curve of animals listed in I. Scale bars A, B, E, F, G = 200 μm, C, D, H = 400 μm.

Having demonstrated that human HER2^+^ breast cancer cells undergo phenotype redirection *in vivo* and *in vitro*, we investigated whether *in vitro* redirection results in a permanent phenotype change. The HER2^+^ cancer cells were co-cultured with MECs for 4 days then magnetically sorted based on HER2 expression. HER2 remains overexpressed on redirected cells and the normal cells do not express HER2; this allows their separation by magnetic sorting. The sorted fractions were then transplanted into cleared mammary fat pads of 3-week old female athymic nude mice. Transplantation of normal MECs resulted in normal mammary ductal tree formation in the recipient animals (Figure 2A and 2B). Transplantation of RFP-expressing cancer cells resulted in the formation of mammary tumors in all instances (4/4) (Figure 2C and 2D). The mammary tumors that formed were comprised entirely of RFP^+^ cells (Figure 2C and 2D). When the HER2^+^ fractions from 1:1 co-cultures of cancer cells and epithelial cells were transplanted normal epithelial growth was found in 75% of the animals in which RFP^+^ cells were also observed (Figure 2E and 2F). Mammary tumors formed in all animals, but the onset of tumor formation was delayed compared to transplantations of tumor cells alone (Figure 2J). When the HER2^+^ fractions from 1:50 co-cultures of cancer cells and epithelial cells were transplanted normal epithelial growth was found in 75% of the animals (Figure 2G and 2I). Many of the ducts contained RFP^+^ cells (Figure 2H). No mammary tumors formed as a result of the transplantation of the HER2^+^ RFP^+^ sorted fractions which had been redirected *in vitro* (Figure 2I). These results suggest that the HER2^+^ breast cancer cells underwent phenotype redirection when co-cultured with breast epithelial cells and the effects of the redirection were maintained during transplantation and subsequent mammary ductal outgrowth.

### *In vitro* redirection induces gene expression profile changes

**Figure 3:**
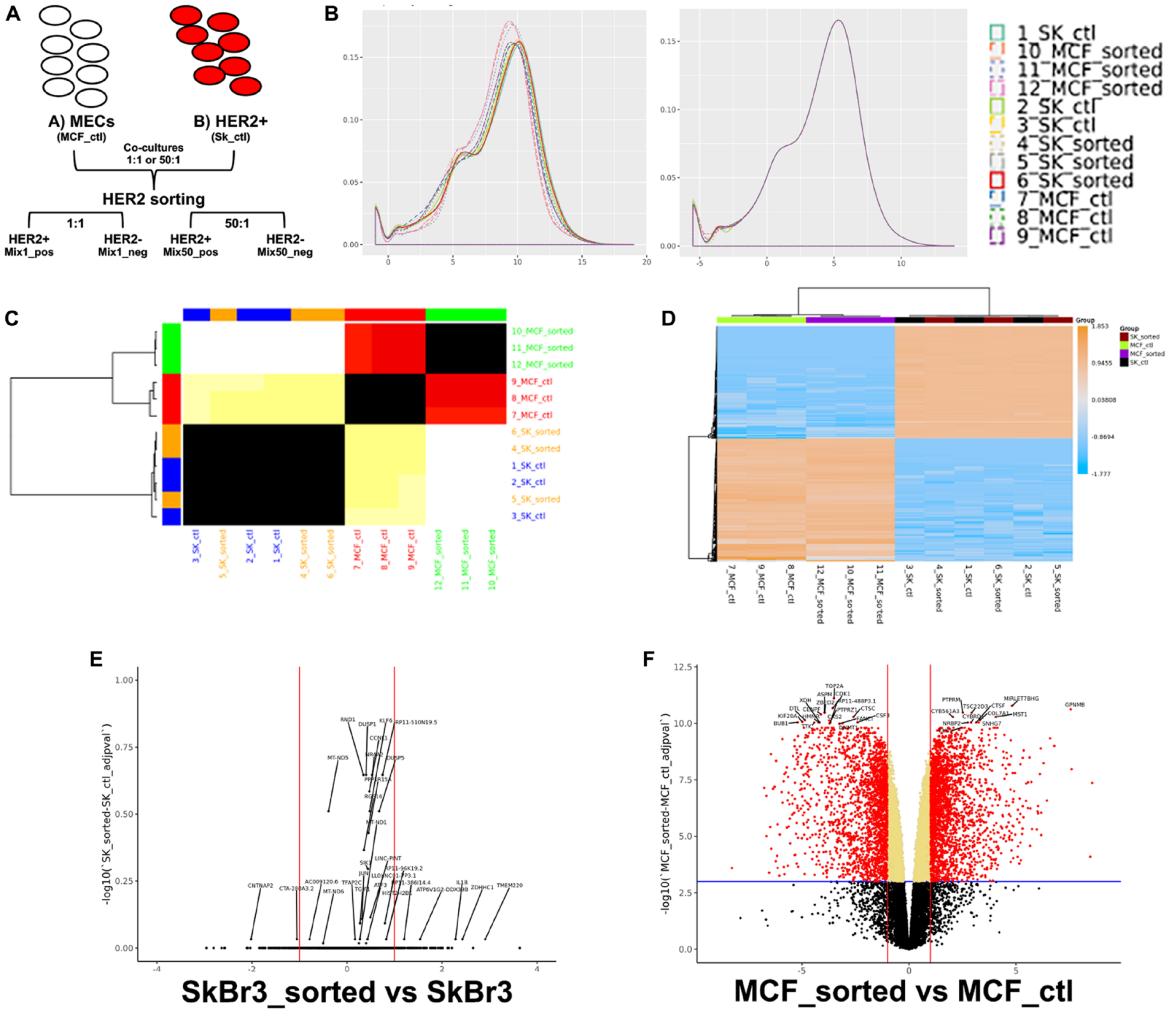
Breast cancer cells express different genes than epithelial cells. (**A**) Schematic describing magnetic sorting approach. (**B**) Effects of quantile normalization; left-before normalization, right-after normalization. (**C**) Similarity heatmap comparing samples of sorted and unsorted cancer and epithelial cells. (**D**) Clustered heatmap of the top 500 most variable genes, by expression, across sorted and non-sorted samples. A color scale bar represents relative gene expression levels within centered rows. (**E**) Volcano plot showing differential expression of 15647 measured genes contrasting sorted and unsorted breast cancer cells. (**F**) Volcano plot showing differential expression of 15647 measured genes contrasting sorted and unsorted epithelial cells. Dashed blue horizontal lines are the adjusted *p*-value threshold (≤ 0.05); dashed red lines are fold change thresholds (fold change ≥ 2.0 or ≤ -2.0). Red dots are genes that passed both thresholds and are those reported as DE in this study.

HER2^+^ cancer cells that undergo redirection, either *in vivo* or *in vitro*, maintain cell surface overexpression of HER2 [3, 6, 16]. By leveraging this we were able to sort co-cultures of HER2^+^ cancer cells and epithelial cells into HER2^+^ and HER2^-^ fractions (Figure 3A). Co-cultures of 1:1 and 1:50 as well as monocultures of HER2^+^ breast cancer cells and breast epithelial cells were subjected to magnetic sorting and the resulting fractions were subjected to RNAseq analysis. After RNA sequencing was performed, data analysis using R/Bioconductor software package *limma* was applied in order to read, normalize the data set, and perform differential expression analyses (Figure 3B). After filtering of low-count genes and quantile normalization, gene expression profiles revealed patterns specific to both cancer cells and epithelial control cells (Figure 3C and 3D). No significant differences were found between gene expression of cancer cells that were or were not subjected to magnetic sorting prior to RNA collection (Figure 3E). However, significant gene expression differences were found between the control epithelial cells that did or did not undergo magnetic sorting prior to RNA collection (Figure 3F). Given this possible sorting effect, and since all RNA collected from co-cultured cells was derived from cells which underwent magnetic sorting, it was decided to only use RNA collected from epithelial cells or cancer cells that underwent magnetic sorting would be used in all further comparisons.

**Figure 4:**
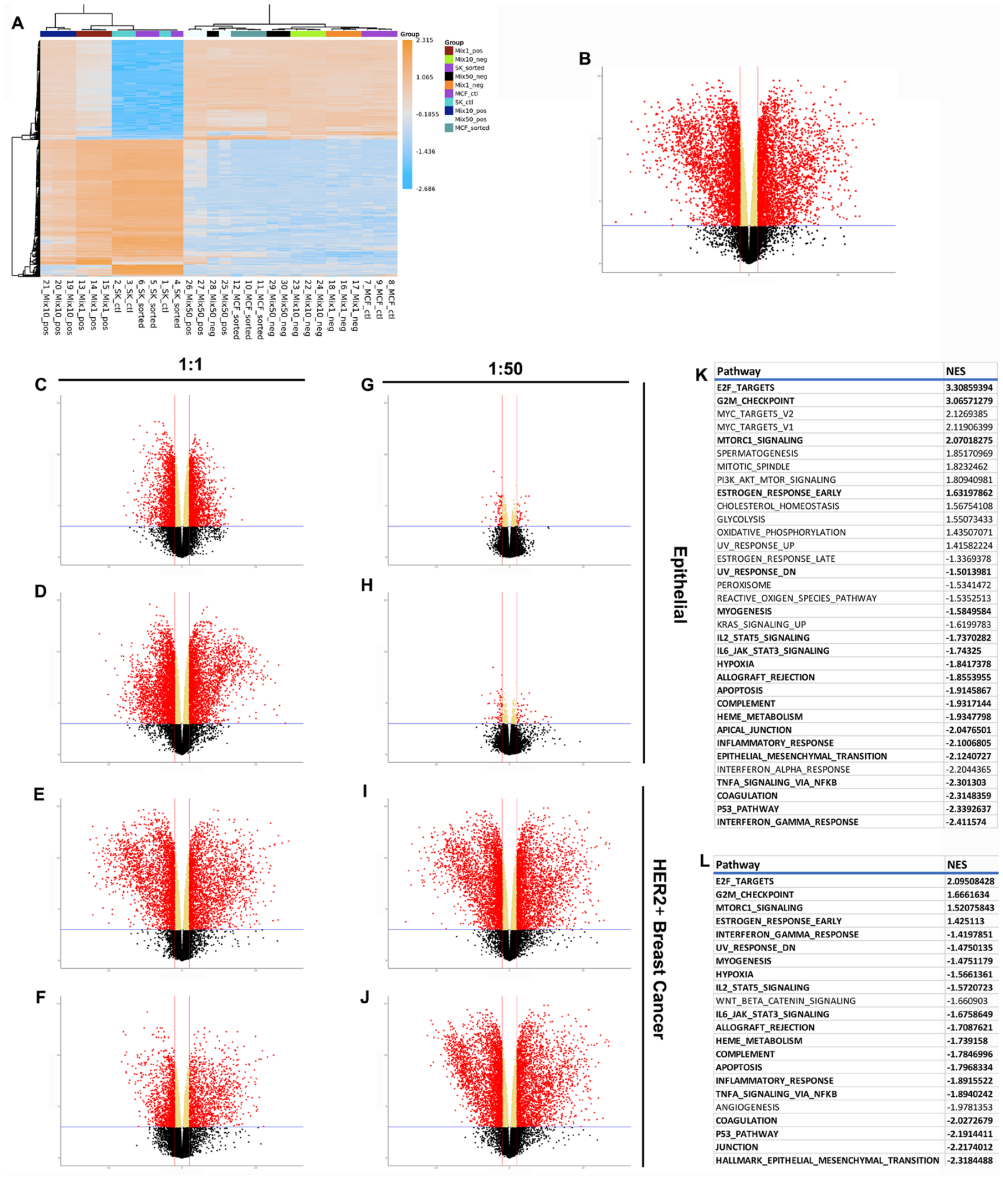
RNAseq results demonstrating differences between normal, cancer, and redirected cells. (**A**) Clustered heatmap of the 500 most variable genes, by expression, across all samples. Volcano plots show differential expression of 15647 measured genes contrasting (**B**) cancer and epithelial cells, (**C**) 1:1 HER2^-^ fraction and epithelial cells, (**D**) 1:1 HER2^+^ fraction and epithelial cells, (**E**) 1:1 HER2^-^ fraction and breast cancer cells, (**F**) 1:1 HER2^+^ fraction and breast cancer cells, (**G**) 1:50 HER2^-^ fraction and epithelial cells, (**H**) 1:50 HER2^+^ fraction and epithelial cells, (**I**) 1:50 HER2^-^ fraction and breast cancer cells, (**J**) 1:50 HER2^+^ fraction and breast cancer cells. Dashed blue horizontal lines are the adjusted *p*-value threshold (≤ 0.05); dashed red lines are fold change thresholds (fold change ≥ 2.0 or ≤-2.0). Red dots are genes that passed both thresholds and are those reported as DE in this study. (**K**) Table of significantly de/activeated pathways between breast cancer cells and epithelial cells. (**L**) Table of significantly de/activated pathways between breast cancer cells and redirected cells. Pathways highlighted in bold appear in both Tables.

When the RNAseq profiles of the six cell combinations are visualized using a heatmap two major distributions are revealed (Figure 4A). One gene distribution consists of the HER2^+^ breast cancer cells cultured alone or with breast epithelial cells in the 1:1 ratio. The second distribution consists of the breast epithelial cells cultured alone, those from the 1:1 and 1:50 co-cultures, and the HER2^+^ cells grown in the 1:50 co-culture. The clustering of the HER2^+^ cells grown in the 1:50 co-culture suggests that they have been redirected *in vitro* and are assuming a normal epithelial gene expression phenotype. When the HER2^+^ profiles are compared to the breast epithelial profile 5614 genes were found to be differentially expressed (DE) (Figure 4B). This result matches our previous findings using mouse mammary tumor cells that over express erbB2, the mouse homolog of HER2 [16].

The comparisons between the normal epithelial cells or the HER2^+^ breast cancer cells and the sorted populations of the co-cultures revealed interesting results. The comparison of the 1:1 HER2^-^ population to control epithelial cells revealed 3673 DE genes (Figure 4C) while 6235 genes were found to be DE in the comparison between the 1:1 HER2^+^ population and the normal epithelial cells (Figure 4D). This difference in DE genes is in line with the observation of 5614 genes DE between the HER2^+^ cancer cells and the normal control cells. Similarly, when the 1:1 HER2^-^ population was compared to the HER2^+^ cancer cells 5629 genes were found DE (Figure 4E) while only 2631 genes were DE in the 1:1 HER2^+^ population vs. HER2^+^ cancer cells comparison (Figure 4F). When the HER2^-^ population was compared to the epithelial control population only 98 genes were found DE indicating that the two populations express essentially the same profile (Figure 4G). However, when the 1:50 HER2^+^ population was equated to the epithelial control population 99 genes were found DE (Figure 4H). The opposite was found when 1:50 populations were matched to the expression profile of the HER2^+^ cancer cells. In both instances, HER2^+^ and HER2^-^, the populations grown in the 1:50 condition had over 6389 and 6428 DE genes compared to the cancer cells respectively (Figure 4I and 4J). These results are consistent with the hypothesis that the HER2^+^ cells have been redirected to adopt a breast epithelial phenotype and move away from a cancer cell phenotype.

Preranked gene set enrichment analysis (GSEA) was performed against the Hallmark collection using the HER2^+^ breast cancer cell dataset and the control breast epithelial dataset [18]. Gene sets with adjusted *p*-values ≤ 0.05 were considered significantly perturbed in cancer samples relative to the controls. In total, 34 gene sets were significantly perturbed in the cancer and control normal groups (Figure 4K). Pre-ranked GSEA was also used to evaluate the gene set differences between the cancer cells and the redirected population. This comparison revealed 22 gene sets to be significantly altered (Figure 4L). The gene sets highlighted in bold appear on both lists. Only 2 of the gene sets listed on the cancer vs redirected comparison are not included in the cancer vs normal epithelial comparison.

## DISCUSSION

Here we investigate a phenomenon we discovered in which cancer cells undergo a switch in phenotype when grown in conjunction with normal mammary epithelial cells either *in vivo* or *in vitro*. We have named this phenotype switching “cancer cell redirection.” Cancer cell redirection was previously shown using mouse mammary cancer-derived cell lines, triple negative breast cancer cell lines, and embryonic carcinoma cells [3–5]. When incorporated into growing ductal trees, stem cell niches direct which phenotype cells will assume during growth [3, 6, 11–16]. Analogous results are shown here for human HER2^+^ breast cancer cells. After being co-cultured with mammary epithelial cells resulting in redirection, the HER2^+^ breast cancer cells lose their tumor forming capacity *in vitro* and give rise to fully developed ductal tree when implanted into cleared mammary fat pads of athymic mice. The differences between the phenotypes of redirected cells and their parent cells are likely due to changes in gene expression levels.

Our data indicates that more than a half of the genes assayed by our RNAseq analysis are significantly DE with absolute fold changes ≥ 2.0 between our breast cancer and epithelial cell lines. Examples of detected DE genes associated with cell-cell interactions include CD44 and TNF-receptors, those involved in apoptotic signaling such as TGF-β, and many others. Elevated expression of these mentioned genes and proteins leads to faster cell proliferation and elimination of apoptotic signaling. In redirected cells, gene expression profiles revert to patterns much more similar to those observed in epithelial cells. HER2^+^-derived redirected cells show very little differences with mammary epithelial (MCF10A) expression profiles; however, HER2^+^-derived redirected cells significantly differ from the original SkBr3 breast cancer cell line.

One notable differentially expressed gene is CD44. CD44 is used as a biomarker for breast cancer stem cells [19]. We found CD44 to be significantly differentially expressed between the cancer cells and epithelial cells (*p* value 1.89E-13) and between the cancer cells and redirected cells (*p* value 1.53E-13). However, there was no significant difference in CD44 expression between the epithelial cells and the redirected cells. CD44 is also included in many of the pathways found to be significantly altered between the cancer cells and epithelial cells and between the cancer cells and redirected cells including the EMT, TNFα_via_NFkB, apoptosis, IL6_JAK_STAT3 and IL2_STAT5 pathways. All five of these pathways are differentially active between the breast cancer cells and both epithelial and redirected cells further indicating that redirected cells are adopting a normal epithelial phenotype. CD44 is known to co-localize with HER2 preventing the binding of the anti-HER2 cancer treatment trastuzumab through steric hinderance [20, 21]. CD44 expression is reduced in redirected cells and redirected cells lose tumor forming ability. Our findings are consistent with the hypothesis that the redirected cancer cells are adopting a normal epithelial phenotype.

Breast cancers are classified as belonging to one of six types of tumors; luminal A, luminal B, basal, claudin-low, normal-like, or HER2 [22]. Of the six classes, only the luminal B and HER2 classes contain HER2^+^ breast cancers. Notably, the HER2^+^ breast cancer cells used in the majority of experiments in this study (SkBr3) are classified as belonging to the HER2 group [23]. We were able to redirect two additional HER2^+^ cell lines. The BT474 cell line is classified as Luminal B [23] while the HCC1954 cell line is classified as basal [24]. Since we are able to redirect multiple breast cancer subtypes, triple negative breast cancer cells [5], and embryonal testicular carcinoma cells [4], this indicates that the phenomenon of cancer cell redirection is not limited to a specific cancer subtype. With additional investigation into other cancer types, cancer cell redirection may provide new targets for the treatment of cancer. If the intracellular pathways involved or the intercellular signals involved can be identified, then they can be modulated.

In conclusion, our data collectively argue that epithelial cells provide signals that influence HER2^+^ breast cancer cells to reduce tumor formation. The reduction in tumor-forming capacity is due to a shift in gene expression profiles from a tumorigenic towards a normal epithelial profile. The phenotypic switch, known as cancer cell redirection, includes changes in the activity of multiple intracellular signaling pathways. Modulation of these affected pathways may be a new approach towards cancer treatments.

## MATERIALS AND METHODS

### Cell culture

BT474 and SkBr3 lines were grown in DMEM with 10% FBS and 1% Pen-Strep. HCC1954 cells were cultured with RPMI media plus 10% FBS and 1% Pen-Strep, while MCF10A cells were grown in DMEM media supplemented with bovine pituitary extract (52 mg/ml), hydrocortisone (0.5 mg/ml), human epidermal growth factor (10 ng/ml), insulin (5 mg/ml), 10% FBS, and 1% Pen-Strep. All cell lines were cultured at 37°C and 5% CO_2_.

### Lentiviral transfection

Lentiviral transfection was performed as previously reported elsewhere [6]. Briefly, all cell lines (MCF10A, BT-474, HCC1954, and SkBr3) were seeded separately into 96-well plates and grown under normal culture conditions (37°C and 5% CO_2_) until 100% confluent. Media was removed and new media without antibiotics was added to the wells. Cultures were grown overnight at 37°C and 5% CO_2_. The cells were washed with PBS, and 30 μl of appropriate media without antibiotics, 20 μl of Cignal Lenti Reporter (Qiagen) or negative control, and 5 μl of SureENTRY Transduction Reagent (Qiagen) were added to each well. BT-474, SkBr3, and HCC1954 cells were transfected with red fluorescent protein protein (RFP) particles, while MCF10A cells were transfected with green fluorescent protein (GFP) particles. Cells were incubated for 24 hours at 37°C and 5% CO_2_. The media was then removed from each well and 100 μl of fresh media containing 500 ng/ml of puromycin was added to select transfected cells in order to generate stable cell lines containing only transfected cells. Puromycin containing media was replaced every 3 days for 12 days, after which it was substituted with cell appropriate media containing 10% FBS and 1% Pen-Strep.

### Magnetic sorting

Cancer cell lines were co-cultured with MCF10A cells in either 1:1 or 1:50 ratios. The co-cultures were then magnetically sorted using Mini and MidiMACS Kits (MACS Miltenyi Biotech). Cells were detached using Trypsin, re-suspended in 500 μL of cold buffer (PBS, pH 7.2, 0.5% BSA, 2 mM EDTA), and centrifuged at 300 × g for 10 minutes. The supernatant was aspirated off, and the cell pellet was re-suspended in 300 μL of cold buffer. 100 μL of cold FcR Blocking Reagent was added to block non-specific binding, and 100 μL of cold anti-ErbB-2 MicroBeads was added. After mixing, the solution was incubated for 30 minutes at 4°C. Cells were washed with 500 μL of buffer, centrifuged at 300 × g for 10 minutes, and re-suspended in 500 μL of buffer. The cell suspension was then passed through a LS Column, and unlabeled cells were collected into a 15 mL centrifuge tube. The LS Column was washed three times with 3 mL of buffer, and the flow-through was added to the unlabeled fraction. A new 15 mL centrifuge tube was placed under the LS Column, the magnet was removed, and 5 mL of buffer was immediately flushed through using a plunger system. The resulting flow-through contained the magnetically labelled cells. Both cell fractions were stored for future implantation or were centrifuged for 5 minutes at 300 × g and re-suspended in 100 μL of RNA *later* for subsequent RNA isolation.

Quality of sorting was determined by flow cytometry, based on presence of fluorescence in the red or green spectrums. Cells were considered successfully transfected only if more then 95% of the culture presented fluorescence.

### Transplantation studies

The transplantation technique used was based on the technique pioneered by DeOme et al. and described elsewhere [8]. Briefly, 3-week old, athymic female mice were anesthetized. The endogenous epithelium of the inguinal mammary glands was removed and 10 μL of cell suspension was injected into the remaining fat pad. After 9 weeks, the animals were euthanized, and mammary outgrowths excised. Half of the samples were prepared for whole mount carmine alum staining and fluorescent observation [25, 26], while the other half was fixed in 10% formalin solution overnight and embedded into paraffin.

The protocols and procedures used to perform the experiments upon the animals were reviewed and approved by the Animal Care and Use Committee of The National Cancer Institute (NCI). Housing and care during the experimental period conformed to the guidelines provided by the National Institutes of Health.

### Histological analysis

Paraffin sections (5 μm) were deparaffinized in xylene and rehydrated in 100% and 95% ethanol then washed with tap water. Rehydrated slides were stained with Hematoxylin and Eosin (H&E) for 5 minutes, dehydrated, and coverslipped.

For fluorescent analysis, sections were deparaffinized and treated with 4′,6-diamidino-2-phenylindole (DAPI) to stain nuclei. Slides were coverslipped using ProLong Gold Antifade mounting medium (Invitrogen).

### Immunostaining

Each cancer cell line (BT474, HCC1954, SkBr3) was mixed in a 1:50 ratio with MCF10A cells in a twelve-well plate containing a cover glass slide and MEGM. Cells were grown to 100% confluency and cultured for four days to allow redirection to occur. Cells were then fixed for 10 minutes with 4% paraformaldehyde and washed 3× for 5 minutes each with PBS. Each cover glass slide was blocked with 10% goat serum for 45 minutes at room temperature. The first primary antibody (anti-ErbB-2) diluted in 1% bovine serum albumin (BSA) solution in PBST (PBS supplemented with 0.1% Tween 20) was applied and incubated for one hour at room temperature. Slides were washed three times in PBS for five minutes, and the first secondary antibody conjugated to Alexa488 in 1% BSA in PBST was applied for 1 hour at room temperature in the dark. Cells were washed three times in PBS for five minutes, and 10% donkey serum was applied and incubated for 30 minutes at room temperature. The second primary antibody (andit-phospho-ErbB2 in 1% BSA/PBST) was incubated for 1 hour at room temperature in the dark, and cells were washed three times with PBS for five minutes. Next, the second secondary antibody conjugated to Alexa568 in 1% BSA/PBST was applied and incubated in the dark for one hour, and the slide was washed three times with PBS for five minutes. Samples were incubated in DAPI for 15 minutes and mounted using Prolong Antifade medium (Invitrogen).

### DNA fragmentation assay

Each cancer cell line (BT474, HCC1954, SkBr3) was mixed in a 1:50 ratio with MCF10A cells in a twelve-well plate containing a cover glass slide and 2–3 mL of mammary epithelial cell growth (MEGM) media. Cells were grown to 100% confluency and cultured for four days to allow redirection to occur. *In situ* BrdU-Red DNA Fragmentation Assay Kit (TUNEL: terminal deoxynucleotidyl transferase dUTP nick end labeling; Abcam) was used according to standard manufacture protocol in order to detect apoptosis. For positive control SkBr3 cells were incubated with 0.2 μg/ml of Doxorubicin in DMEM overnight.

### RNA isolation and RNAseq

Total RNA was isolated using RNAqueous Micro Kits (Ambion, Austin, TX) that utilize purification achieved through glass fiber column filtration. Possible DNA contamination was removed from isolated RNA by treating the samples with DNase (Qiagen, Valencia, CA). The concentration of RNA was determined using a NanoDrop and the RNA integrity was analyzed with an RNA nanochip on a Bioanalyzer (Agilent Technologies, Santa Clara, CA).

Samples were pooled and sequenced via mRNA-seq (10 different groups with three repeats each) on NextSeq using Illumina TruSeq Stranded mRNA Library Prep and paired-end sequencing. Obtained reads were trimmed of low-quality bases and adapter sequences were removed using Cutadapt v1.18 [27]. Mapping of reads to the GRCh38 (hg38) human reference genome was performed using STAR v2.6.1 in 2-pass mode [28]. Then, RSEM v1.2.31 was used to quantify gene-level expression, with counts normalized to library size as counts-per-million [29]. Finally, limma-voom v3.34.5 was used for quantile normalization and differential expression of genes analysis [30]. Genes that were both significantly differentially expressed relative to control (adjusted *p* < 0.001) and that had absolute fold changes relative to control ≥ 2.0 were retained for further analysis.

Pathway Overrepresentation analysis (using Fisher’s Exact Test) against the Hallmarks collection was performed. Results were filtered based on adjusted *p*-value then sorted on Normalized Enrichment Score (NES).

### Statistics

All numerical data is presented as the mean value ± standard deviation. One-way ANOVA was used for variance comparison. Repeated measures ANOVA was used for the analysis of image intensity for fluorescent microscopy. Statistical difference was considered significant if *P*-value was less than 0.05.
