# Correction: Induction of phenotypic changes in HER2-postive breast cancer cells *in vivo* and *in vitro*

**DOI:** 10.18632/oncotarget.28520

**Published:** 2023-09-28

**Authors:** Anastasia Frank-Kamenetskii, Julia Mook, Meredith Reeves, Corinne A. Boulanger, Thomas J. Meyer, Lauren Ragle, H. Caroline Jordan, Gilbert H. Smith, Brian W. Booth

**Affiliations:** ^1^Department of Bioengineering, Clemson University, Clemson, SC, USA; ^2^Department of Biological Sciences, Clemson University, Clemson, SC, USA; ^3^Center for Cancer Research, National Cancer Institute, National Institutes of Health, Bethesda, MD, USA; ^4^CCR Collaborative Bioinformatics Resource, National Cancer Institute, National Institutes of Health, Bethesda, MD, USA; ^5^Advanced Biomedical Computational Science, Frederick National Laboratory for Cancer Research, Frederick, MD, USA; ^*^These authors contributed equally to this work

**This article has been corrected:** In Figure 2, panels A and B contain accidental ‘placeholder’ images that contain partial duplications of panels E and G. The corrected figure 2, obtained using the original data, shown below. The authors declare that these corrections do not change the results or conclusions of this paper.

Original article: Oncotarget. 2020; 11:2919–2929. 2919-2929. https://doi.org/10.18632/oncotarget.27679

**Figure 2:**
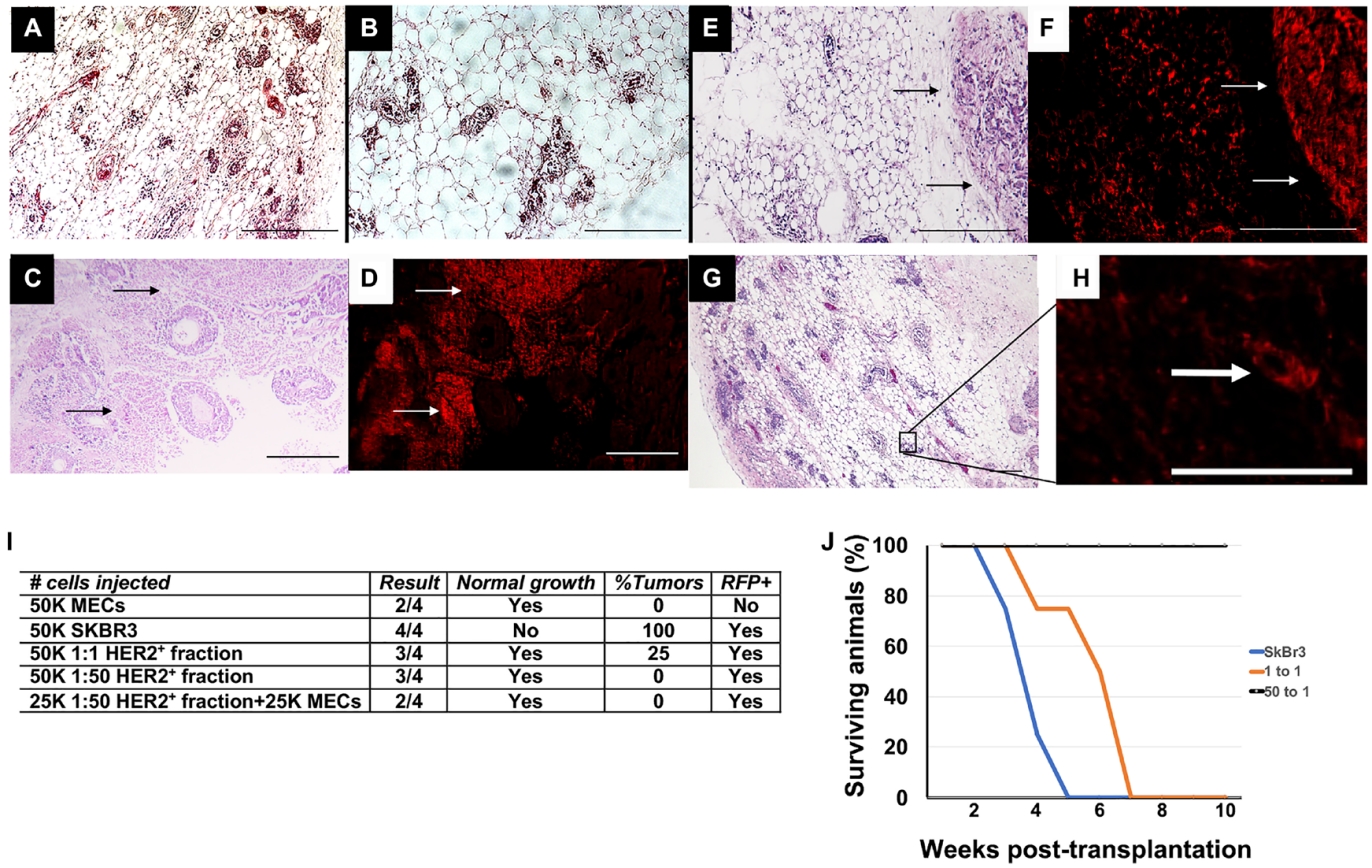
Transplantation results following *in vitro* redirection. (**A**, **B**) H&E staining of mammary outgrowth following MEC transplantation. (**C**) H&E staining of mammary tumor that formed following transplantation of SkBr3-RFP cells. (**D**) Fluorescent image of C. (**E**) H&E staining of mammary outgrowth and mammary tumor following transplantation of HER2+ 1:1 fraction. (**F**) Fluorescent image of E. (**G**) H&E staining of HER2^+^ 1:50 fraction. (**H**) Fluorescent image of outlined area in G. (**I**) Transplantation results. (**J**) Survival curve of animals listed in I. Scale bars A, B, E, F, G = 200 μm, C, D, H = 400 μm.

